# CeO_2_ Nanoparticles-Regulated Plasmid Uptake and Bioavailability for Reducing Transformation of Extracellular Antibiotic Resistance Genes

**DOI:** 10.3390/nano13060969

**Published:** 2023-03-08

**Authors:** Yinuo Xu, Hao Du, Chuanxi Wang, Le Yue, Feiran Chen, Zhenyu Wang

**Affiliations:** 1 Institute of Environmental Processes and Pollution Control, and School of Environmental and Civil Engineering, Jiangnan University, Wuxi 214122, China; 2 Jiangsu Engineering Laboratory for Biomass Energy and Carbon Reduction Technology, Jiangnan University, Wuxi 214122, China; 3 Jiangsu Collaborative Innovation Center of Technology and Material of Water Treatment, Suzhou University of Science and Technology, Suzhou 215009, China

**Keywords:** plasmid-borne ARGs, CeO_2_ nanoparticles, transformation, regulation, binding and uptake

## Abstract

The direct uptake of extracellular DNA (eDNA) via transformation facilitates the dissemination of antibiotic resistance genes (ARGs) in the environment. CeO_2_ nanoparticles (NPs) have potential in the regulation of conjugation-dominated ARGs propagation, whereas their effects on ARGs transformation remain largely unknown. Here, CeO_2_ NPs at concentrations lower than 50 mg L^−1^ have been applied to regulate the transformation of plasmid-borne ARGs to competent *Escherichia coli* (*E. coli*) cells. Three types of exposure systems were established to optimize the regulation efficiency. Pre-incubation of competent *E. coli* cells with CeO_2_ NPs at 0.5 mg L^−1^ inhibited the transformation (35.4%) by reducing the ROS content (0.9-fold) and cell membrane permeability (0.9-fold), thereby down-regulating the expression of genes related to DNA uptake and processing (*bhsA*, *ybaV*, and *nfsB*, 0.7–0.8 folds). Importantly, CeO_2_ NPs exhibited an excellent binding capacity with the plasmids, decreasing the amounts of plasmids available for cellular uptake and down-regulating the gene expression of DNA uptake (*bhsA*, *ybaV*, and *recJ*, 0.6–0.7 folds). Altogether, pre-exposure of plasmids with CeO_2_ NPs (10 and 25 mg L^−1^) suppressed the transformation with an efficiency of 44.5–51.6%. This study provides a nano-strategy for controlling the transformation of ARGs, improving our understanding on the mechanisms of nanomaterial-mediated ARGs propagation.

## 1. Introduction

The abuse and misuse of antibiotics make antimicrobial resistance one of the biggest threats to global health and food security [1], and the extensive use of antibiotics during COVID-19 pandemic has worsened the situation [2]. The horizontal gene transfer (HGT) of antibiotic resistance genes (ARGs) is one of the primary drivers for antimicrobial resistance propagation [3]. HGT happens via conjugation, transformation, transduction, and additional processes involved in outer membrane vesicles [4]. The initiation of conjugation or transduction requires the conjugative pilus or phage to transfer ARGs carried by intracellular DNA, and transformation involves a direct uptake of extracellular DNA (eDNA, e.g., plasmids) by competent bacteria [5]. Although conjugation has been considered as the primary process of HGT and research has focused on the inhibition of intracellular DNA propagation [6], emerging studies have addressed the importance of eDNA dissemination dominated by transformation. In marine environments, the frequency of transformation (10^−6^–10^−7^ resistant cells per total cell count) is higher than those of conjugation (around 10^−7^) and transduction (10^−7^–10^−9^) [7,8,9]. Consistently, eDNA occurs at a concentration over 10–70 fold higher than that of intracellular DNA in marine sediments [10]. Besides, eDNA in soil and sediments is abundant and relatively stable (retention for months) due to its adsorption on soil colloids, sand particles, and clay minerals [11,12]. On this basis, it is urgent to develop strategies to inhibit the transformation of eDNA, thus alleviating the spread of ARGs.

Transformation of eDNA includes the development of cell competence, eDNA binding to cell membrane, uptake, and processing [13]. The competence of recipient bacteria is a prerequisite for transformation to take place [5]. The cell membrane serves as a natural barrier against internalization of eDNA [14]. Inhibition of the synthesis of cell membrane and an increase in cell permeability promotes the transformation-mediated gene transfer. The closely packed phospholipids and lipopolysaccharides are essential for the permeability of bacterial membrane [15]. Overaccumulation of reactive oxygen species (ROS) can attack lipopolysaccharides, leading to an increase of membrane permeability [16,17,18]. Besides, the incorporation of eDNA is affected by its availability. Binding of eDNA with natural particles (e.g., montmorillonite, illite, and sand) prevents the degradation and facilitates the persistence of eDNA in the environment [19]; however, this binding may reduce the amount of eDNA available for transformation [20]. Nanomaterials have been introduced as a potential alternative for controlling the conjugation transfer of iDNA [7], but their roles in the transformation of eDNA remains largely unknown. The adsorption of eDNA to the nanoparticles (NPs) was irreversible, decreasing the bioavailability of eDNA and inhibiting bacterial growth [21,22]. In addition, NPs might interact with the cell membrane upon direct contact and NPs with ROS scavenging capacity may contribute to the maintenance of membrane permeability [23,24]. CeO_2_ NPs are known as effective ROS scavengers and can remove excess ROS (by 52%) in *Arabidopsis thaliana* without nano-toxicity [25,26]. A CeO_2_ adsorbent exhibited a high adsorption capacity for the removal of ARGs (eDNA), with a maximum adsorption efficiency up to 83.4% [27]. Considering the ROS scavenging and the potential binding capacities of CeO_2_ NPs, we hypothesize that CeO_2_ NPs may inhibit transformation through eDNA binding, eliminating ROS, and maintaining cell membrane permeability.

To investigate the roles of CeO_2_ NPs on plasmid binding and on membrane permeability, three transformation systems were established to identify the optimal stage and concentration of CeO_2_ NPs for controlling the transformation of plasmid-borne ARGs. *Escherichia coli* (*E. coli*) was used as a recipient strain, considering its frequent occurrence in manure and biosolids; it is also known to be persistently (≤231 days) competent in soils [28]. pAC plasmids at 4–6 × 10^9^ copies mL^−1^, equivalent to the concentration reported in sludge, livestock manure, and household wastewater [7,29], were applied to the transformation systems. The interactions between plasmids and CeO_2_ NPs, ROS content, cell membrane permeability, and expression of genes involved in the transformation process were measured and analyzed to reveal the CeO_2_ NPs-mediated mechanisms. Our findings could advance the understanding of CeO_2_ NPs-regulated transformation of ARGs, providing an alternative approach for the management of antibiotic resistance in the environment.

## 2. Materials and Methods

### 2.1. Plasmid Extraction, Bacterial Strains and Characterization of CeO_2_ NPs

The pAC plasmids (3184 bp), marked with *mCherry* fluorescent protein carrying a chloramphenicol resistance gene (*Cm^R^*), were purchased from GenScript Biotech Corporation (Nanjing, China). *E. coli* cells harboring the pAC plasmids were incubated in Luria-Bertani (LB) medium (Hopebiol, Qingdao, China) containing 10 μg mL^−1^ chloramphenicol at 37 ℃ overnight on a shaker (220 rpm). The plasmids were extracted with SanPrep Column Plasmid Mini-Preps Kit (Sangon Biotech, Shanghai, China), and the concentration and quality (A_260_/A_280_ ratio > 1.8) of plasmids were determined using a Nanodrop spectrophotometer (Gallop Technology, Shanghai, China).

*E. coli* JM109 strain, a non-resistant bacterium, was selected as the recipient strain to assess the efficiency and frequency of plasmid transformations. For the transformation of plasmid-borne ARGs into *E. coli* cells, an Ultra-Competent Cell Preps Kit (Sangon Biotech, Shanghai, China) was used to ensure *E. coli* JM109 was competent for transformation.

CeO_2_ NPs (99.99%, 10 nm) were purchased from Sigma-Aldrich (Newburyport, MA, USA). CeO_2_ NPs suspensions (1 mg mL^−1^) were prepared by dispersing 0.5 g of CeO_2_ NPs in 50 mL sterile Milli-Q water (Millipore, Burlington, MA, USA) via ultrasonication (80 W) for 1 h in a water bath at ambient temperature, followed by dilution to working concentrations in a range of 0–50 mg L^−1^ for subsequent experiments. The morphology of CeO_2_ NPs was observed by transmission electron microscopy (TEM, JEM-2100, JEOL, Akishima, Japan, Figure S1a). The diameter is around 20 nm. The structure of CeO_2_ NPs was investigated by X-ray diffraction (XRD, D2 Phaser, Bruker AXS, Karlsruhe, Germany, Figure S1b), indicating a composition of pure cubic cerium oxide. The hydrodynamic diameter and zeta-potential were measured using a Zetasizer (Nano-ZS90, Malvern Instruments, Malvern, UK, Table S1). To assess the dissolution of CeO_2_ NPs, CeO_2_ NPs suspensions (0, 0.5, 1, 10, 25, and 50 mg L^−1^) were prepared and placed for 2 h followed by filtration using Amicon Ultra 3 kDa MWCO tubes (2348× *g* for 15 min). The Ce^3+^ content in supernatants was analyzed using inductively coupled plasma mass spectrometry (ICP-MS, iCAP-TQ, Thermo Fisher, Karlsruhe, Germany).

### 2.2. Establishment of Transformation Systems and Measurement of Transformation Efficiency

Three transformation systems were set up by using pAC plasmids as extracellular plasmid-borne ARGs and competent *E. coli* cells as recipients. To investigate the roles of CeO_2_ NPs on the process of transformation, CeO_2_ NPs were incubated with plasmids or *E. coli* alone or in combination.

System 1 was established by mixing plasmids and *E. coli* cells with CeO_2_ NPs. Briefly, 25 ng free plasmids and competent *E. coli* cells (10^7^ cfu mL^−1^) were exposed to CeO_2_ NPs at concentrations of 0, 0.5, 1, 5, 10, 25, and 50 mg L^−1^ (100 μL transformation system), respectively. The mixture was placed in an ice-water bath and incubated for 2.5 h.

System 2 was conducted by pre-incubation of *E. coli* with CeO_2_ NPs followed by an addition of plasmids for transformation. Specifically, 100 μL competent *E. coli* cells (10^7^ cfu mL^−1^) were exposed to CeO_2_ NPs at concentrations of 0, 0.5, 1, 5, 10, 25, and 50 mg L^−1^, respectively. After 2 h exposure in an ice-water bath, 25 ng plasmids were added into the mixture, followed by 30 min of incubation in an ice-water bath.

System 3 was carried out by pre-incubation of plasmids with CeO_2_ NPs followed by an addition of *E. coli* cells. Briefly, 25 ng pAC plasmids were exposed to CeO_2_ NPs at concentrations of 0, 0.5, 1, 5, 10, 25, and 50 mg L^−1^, respectively. After incubation on ice for 2 h, the mixture was transferred into 100 μL suspensions of competent *E. coli* (10^7^ cfu mL^−1^) and incubated in an ice-water bath for 30 min.

In all systems, after exposure to CeO_2_ NPs, each mixture was heat-shocked at 42 °C for 90 s. After resting in the ice-water bath for 3 min, the bacteria suspensions were incubated in 900 μL LB medium at 37 °C, 220 rpm for 1 h. The amount of total culturable cells was calculated by plating samples on LB agar plates containing no antibiotics. Transformants were selected by dispersing another 100 μL of bacterial suspensions on LB agar plates containing 10 μg mL^−1^ chloramphenicol. The transformation efficiency and transformation frequency were calculated with the following formulas [20]:

Transformation efficiency = number of transformants (cfu)/pAC plasmids (ng)

Transformation frequency = number of transformants (cfu)/total amount of culturable *E. coli* cells (cfu)

### 2.3. Identification of Transformants

To confirm whether the transformants on antibiotic-containing plates carried the target pAC plasmids, colonies were randomly chosen and cultured in LB medium overnight. Plasmids were extracted using the SanPrep Column Plasmid Mini-Preps Kit (Sangon Biotech, Shanghai, China). The target gene containing both *Cm^R^* and *mCherry* (2651 bp) was detected with long amplicon PCR (Bio-Rad C1000 Touch, Hercules, CA, USA) (primers used are listed in Table S2). Each amplicon PCR reaction contained 25 µL of PrimeSTAR Max DNA Polymerase (Takara, Beijing, China), 2 µL of forward and reverse primers (10 μM each), 1 µL of DNA templates, and 20 µL of sterile double distilled H_2_O. The presence of ARGs was identified using agarose gel electrophoresis with a band in a size of 2000–3000 bp (Figure S2).

### 2.4. FTIR Analysis and Quantification of Plasmids after Binding with CeO_2_ NPs

To characterize the binding of plasmids with CeO_2_ NPs, 25 ng pAC plasmids were exposed to CeO_2_ NPs (0, 0.5, 1, 5, 10, 25, and 50 mg L^−1^, respectively), followed by incubation on ice for 2 h. The mixture was dropwise loaded on the crystal surface of attenuated total reflectance (ATR) attachment for FTIR analysis (Nicolet iS20, Thermo Scientific, USA). CeO_2_ NPs-bound plasmids were separated from free plasmids by centrifugation at 9391× *g* and 25 °C for 10 min. The loss rate of plasmids after 2 h exposure was determined using a NanoDrop spectrometer and calculated with the following formula:

Loss rate = (plasmid concentration_0h_ (ng μL^−1^) − plasmid concentration_2h_ (ng μL^−1^))/plasmid concentration_0h_ (ng μL^−1^)

### 2.5. Measurements of ROS and Cell Membrane Permeability

The competent *E. coli* cells were incubated with 10 μM of 2′,7′-dichlorofluorescein diacetate (DCFDA, Sigma-Aldrich, St. Louis, MO, USA) for 30 min at 37 °C in darkness, with gentle shaking every 5 min. The mixture was washed three times with PBS to remove extra DCFDA. Afterwards, the bacteria cells were exposed to CeO_2_ NPs as described in System 1–3. The fluorescence intensity of each sample was measured using a microplate reader (Thermo Scientific, Waltham, MA, USA) with an excitation wavelength of 488 nm and an emission wavelength of 525 nm [30].

For the measurement of cell membrane permeability, the competent *E. coli* cells sampled from System 1–3 were incubated with 0.5 μM propidium iodide (PI, Beyotime, Shanghai, China) dye for 15 min at 37 °C in darkness. The fluorescence intensity was measured by a microplate reader with an excitation wavelength of 535 nm and an emission wavelength of 615 nm [31].

### 2.6. Measurement of CeO_2_ NPs Internalized by E. coli Cells

The competent *E. coli* cells exposed to CeO_2_ NPs or CeO_2_ NPs combined with plasmids were washed three times with PBS and centrifuged at 2348× *g*, 25 °C for 20 min. The collected bacteria were transferred into a 5 mL centrifuge tube for enzymatic digestion with 3 mg L^−1^ pancreatin mixed with 3 g L^−1^ lipase in PBS. After incubation for 12 h at 35 °C, the supernatant was obtained by centrifugation (1006× *g*, 15 min) [32]. The supernatant was filtered (0.22 μm) and diluted, and the number of NPs was measured by single particle-ICP-MS.

### 2.7. Relative Expression of Transformation-Related Genes Determined by qRT-PCR

The total RNA of bacteria was extracted using RNApure Bacteria Kit (CWBIO, Beijing, China) according to the manufacturer’s instructions and quantified using a Nanodrop spectrometer. Reverse transcription of extracted RNA was performed using the EasyQuick RT MasterMix (CWBIO, Beijing, China) following the manufacturer’s protocol. Primers used are listed in Table S2. RT-PCR analysis was performed using a Bio-Rad CFX96 touch instrument (Bio-Rad, Hercules, CA, USA) with UltraSYBR mixture (CWBIO, Beijing, China) under the following program: predenaturation step at 95 °C for 30 s, 40 cycles of denaturation for 5 s at 95 °C, primer annealing for 30 s at 60 °C. The relative gene expression was calculated using a 2^−ΔΔCT^ method [33].

### 2.8. Statistical Analysis

All experiments were performed with three biological replicates, and each biological replicate involved three technical replicates. Data are presented as mean ± standard deviation, and the significant differences among treatments were tested by one-way analysis of variance (ANOVA) tests followed by a least significant difference (LSD) test where a *p* < 0.05 was considered statistically significant.

## 3. Results and Discussion

### 3.1. Effects of CeO_2_ NPs on the Transformation of eDNA

**Figure 1:**
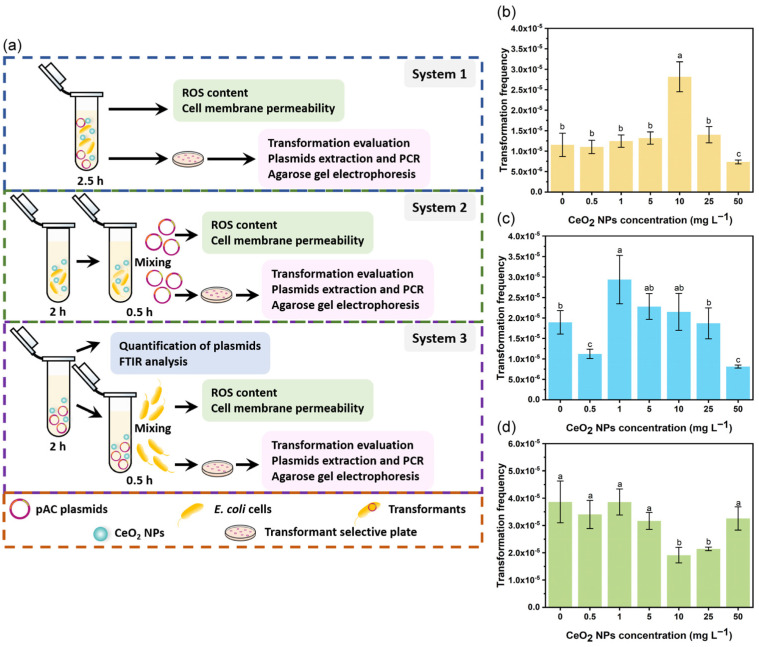
Effects of CeO_2_ NPs on the transformation frequency of extracellular ARGs. Schematic diagram of transformation systems (**a**). Transformation frequency of pAC plasmid in System 1 (**b**), System 2 (**c**), and System 3 (**d**). Lowercase letters represent the statistical significance among different concentrations of CeO_2_ NPs.

Incubation of plasmids and competent *E. coli* cells with CeO_2_ NPs (System 1, Figure 1a) at 0.5–5 mg L^−1^ did not affect the transformation, while 10 mg L^−1^ of CeO_2_ NPs dramatically increased the transformation frequency by 144.1% (Figure 1b). However, the transformation frequency decreased by 36.5% when the concentration of CeO_2_ NPs increased to 50 mg L^−1^ (Figure 1b). In System 2 (Figure 1a), incubation of competent *E. coli* cells with CeO_2_ NPs at 0.5 or 50 mg L^−1^ significantly inhibited (by 35.4–57.1%) the transformation, while 1 mg L^−1^ of CeO_2_ NPs distinctively promoted (by 55.6%) the transformation (Figure 1c). Correspondingly, the transformation efficiency reduced by 24.1% and 67.7% after incubation with 0.5 or 50 mg L^−1^ CeO_2_ NPs, respectively (Figure S3). Unlike System 1 and 2, no promoting effect of CeO_2_ NPs on transformation was observed in System 3, where plasmids were pre-incubated with different concentrations of CeO_2_ NPs. Notably, the transformation frequency decreased by 51.6% or 44.5%, and the transformation efficiency decreased by 42.3% or 37.6% in the presence of 10 or 25 mg L^−1^ CeO_2_ NPs, respectively (Figure 1d; Figure S3).

**Figure 2:**
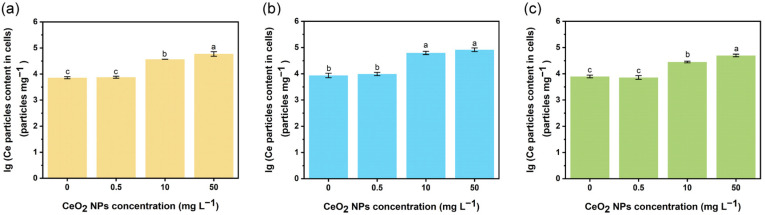
Uptake of CeO_2_ NPs by *E. coli* cells after exposure. Particle content CeO_2_ NPs internalized in *E. coli* cells in System 1 (**a**), System 2 (**b**) and System 3 (**c**). Lowercase letters represent the statistical significance among different concentrations of CeO_2_ NPs.

Considering the various effects of CeO_2_ NPs at different concentrations, the concentration of CeO_2_ particles internalized in *E. coli* cells were measured using single particle-ICP-MS (Figure 2). In general, the size and concentration increased in a concentration-dependent manner in all three systems. The least amount of CeO_2_ particles (28,317.2 ± 2060.0 particles mg^−1^ and 50,184.4 ± 5645.1 particles mg^−1^ for 10 and 50 mg L^−1^, respectively) and the smallest particle size were observed in System 3 (average size 35.6–37.2 nm), while the maximum amount of CeO_2_ particles was detected in System 2 (62,475.8 ± 8699.1 particles mg^−1^ and 82,151.0 ± 12,575.9 particles mg^−1^ for 10 and 50 mg L^−1^, respectively, Figure 2 and Table S3). The low concentration and small size of internalized CeO_2_ particles in System 3 might be attributed to the interaction of plasmids with CeO_2_ NPs. Pre-incubation of competent *E. coli* cells with CeO_2_ NPs (1 or 10 mg L^−1^) may promote the transformation process, while CeO_2_ NPs at 50 mg L^−1^ can inhibit the transformation regardless of the presence of plasmids. Pre-incubation of plasmids with CeO_2_ NPs at 10 or 25 mg L^−1^ may reduce the bioavailability of plasmids; this will be discussed in the following section. High concentrations of CeO_2_ NPs might attach to the surface of *E. coli* cell after the incubation, and a small part of CeO_2_ NPs entered the bacterial cell via endocytosis. Particles with a positive charge will bind to the negatively charged cell surface and 20–50 nm size particles are taken up more rapidly than smaller or larger particles [34].

### 3.2. Interactions between Plasmid and CeO_2_ NPs

**Figure 3:**
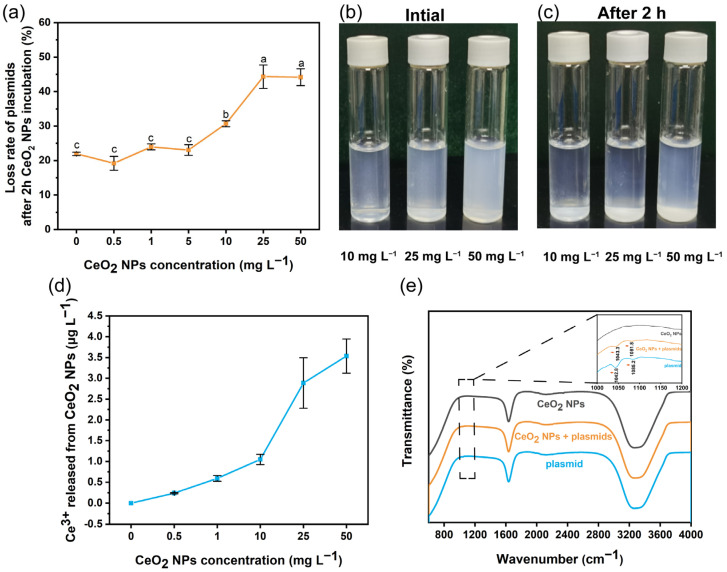
Interactions between plasmids and CeO_2_ NPs. Loss rate of pAC plasmids after incubation with CeO_2_ NPs for 2 h (**a**). Images of CeO_2_ NPs self-aggregation at 10, 25, and 50 mg L^−1^ for 2 h (**b**,**c**). Dissolution of CeO_2_ NPs (**d**). Comparison of FTIR spectra of pAC plasmids before and after incubation with CeO_2_ NPs (**e**). Lowercase letters represent the statistical significance among different concentrations of CeO_2_ NPs.

**Figure 4:**
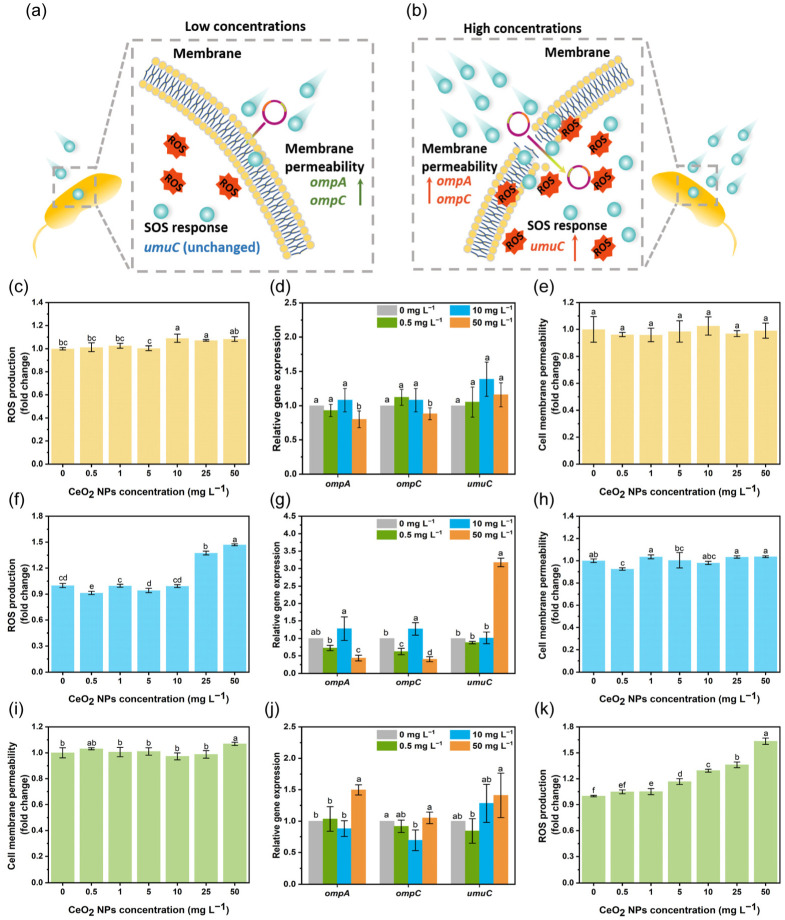
Effect of CeO_2_ NPs on the ROS generation and cell membrane permeability. Illustrative diagrams of ROS, membrane permeability, SOS response, and corresponding gene expressions regulated by CeO_2_ NPs exposure (**a**,**b**). Fold changes of ROS content regulated by CeO_2_ NPs in System 1 (**c**). Relative gene expression in System 1 (**d**). Fold changes of cell membrane permeability regulated by CeO_2_ NPs in System 1 (**e**). Fold changes of ROS content regulated by CeO_2_ NPs in System 2 (**f**). Relative gene expression in System 2 (**g**). Fold changes of cell membrane permeability regulated by CeO_2_ NPs in System 2 (**h**). Fold changes of cell membrane permeability induced by CeO_2_ NPs in System 3 (**i**). Relative gene expression in System 3 (**j**). Fold changes of ROS content induced by CeO_2_ NPs in System 3 (**k**). Lowercase letters represent the statistical significance among different concentrations of CeO_2_ NPs.

Free eDNA is susceptible to intermolecular aggregation, reducing the chance of HGT in the environment [35]. The binding of extracellular plasmids with NPs can decrease the number of plasmids entering the bacteria [20]. The loss rate of plasmids after pre-exposure to CeO_2_ NPs at low concentrations (0.5–5 mg L^−1^) remained relatively low and was similar to that of control group (18.9–24.3%, Figure 3a); high concentrations (10, 25, and 50 mg L^−1^) elevated the loss rate to 30.7–44.3% (Figure 3a). Consistently, the increased loss of plasmids may contribute to the decreased transformation frequency observed in System 3 (by 51.6% and 44.5% at 10 and 25 mg L^−1^, respectively) (Figure 1d). However, the transformation frequency of plasmids exposed to 50 mg L^−1^ CeO_2_ NPs remained unchanged (Figure 1d). The loss rate of plasmids after 50 mg L^−1^ exposure was similar to that of 25 mg L^−1^ exposure (Figure 3a); this could be attributed to the self-aggregation of CeO_2_ NPs at high concentration. The initial hydrodynamic diameter of CeO_2_ NPs suspensions at 10, 25, and 50 mg L^−1^ was 1173.5 ± 69.6 nm, 1117.9 ± 143.6 nm, and 1278.7 ± 64.6 nm, respectively; and the diameter increased to 1177.2 ± 96.7 nm, 1140.7 ± 75.3 nm, and 2349.4 ± 243.1 nm, respectively, after 2 h (Table S4), indicating a strong self-aggregation of CeO_2_ NPs at 50 mg L^−1^ (Figure 3b,c). Aggregation can decrease the specific surface area, physically blocking the reactive sites on particles [36]. After incubating plasmids with CeO_2_ NPs (System 3), the hydrodynamic diameter reached 1386.3 ± 82.9 nm, 1437.2 ± 146.5 nm, and 2046.4 ± 173.9 nm for CeO_2_ NPs exposure at 10, 25, and 50 mg L^−1^, respectively (Table S4). The significant increase (by 17.8% or 26.0%) in hydrodynamic diameter indicated an aggregation of plasmids with CeO_2_ NPs at 10 or 25 mg L^−1^, while 50 mg L^−1^ of CeO_2_ NPs decreased the hydrodynamic diameter of CeO_2_ NPs-treated plasmids (by 12.9%). Thus, it seems that the self-aggregation of CeO_2_ NPs (50 mg L^−1^) may reduce the reactive sites of NPs for plasmid binding. Moreover, the increased membrane permeability resulted from 50 mg L^−1^ CeO_2_ NPs exposure (Figure 4) may elevate the transformation frequency back to the control level.

It is known that DNA molecules can condense into compact structures when the valency of counter-ions ≥ three [35]. However, Ce ions did not contribute to the CeO_2_ NPs-plasmids aggregation as almost no dissolution (<0.1%) was observed for CeO_2_ NPs suspensions (ranging 0.5–50 mg L^−1^, Figure 3d). This extremely low dissolution might relate to the neutral reaction solution [37]. The FTIR spectra confirm the interaction between CeO_2_ NPs and plasmids (System 3). CeO_2_ could act as a Lewis acid and bind with Lewis bases, such as phosphate backbone or electron-rich aromatic rings [38]. Nanomaterials containing transition metallic f-element (e.g., Ce) on the surface are likely to form strong interactions with the phosphate backbones of ARGs [39,40]. Thus, the possible binding site might be the phosphate backbone of DNA or π-system of the purine or pyrimidine aromatic ring. Besides, the absorption bands of CeO_2_ NPs-exposed plasmids (P-O and C-O) at 1042.8 cm^−1^ shifted slightly to 1042.3 cm^−1^ and became weaker; the band at 1085.2 cm^−1^ shifted to 1081.8 cm^−1^ (Figure 3e). The shift of absorption bands indicated a binding of plasmids with CeO_2_ NPs via phosphate groups. Besides, the zeta-potential of plasmids was −6.6 ± 0.9 mV; this negative charge could be attributed to the phosphate group, indicating a potency to bind positively charged molecules [41]. The zeta-potential of CeO_2_ NPs at 10, 25, and 50 mg L^−1^ was 24.7 ± 0.5 mV, 23.9 ± 0.8 mV, and 24.2 ± 1.8 mV, respectively, suggesting an aggregation with plasmids via electrostatic attraction. Thus, pre-exposure of plasmids with CeO_2_ NPs (10 and 25 mg L^−1^, System 3) can reduce the availability of plasmids for transformation.

### 3.3. Regulation of ROS Generation and Cell Membrane Permeability by CeO_2_ NPs

Oxidative stress due to overaccumulation of ROS is one of the primary drivers for the development of antimicrobial resistance [42]. Overaccumulation of ROS may increase membrane permeability and activate the SOS response [43]. Activation of the SOS response enables the uptake of eDNA by competent strains for repairing or developing antibiotic resistance [44]. Bacterial cell membrane is known as a barrier against HGT, inhibiting the transfer of eDNA into cells [45,46] (Figure 4a,b). Our previous work showed that CeO_2_ NPs at low concentrations exhibited an efficient ROS scavenging capacity [47]. In System 1, although CeO_2_ NPs at 10–50 mg L^−1^ increased the ROS level by 1.1-fold, (Figure 4c), the cell membrane permeability remained unchanged for all concentrations, and was confirmed by the expression pattern of genes related to membrane permeability and the SOS response (Figure 4d,e). Incubation of competent *E. coli* cells with CeO_2_ NPs in System 2 at 0.5 mg L^−1^ reduced the ROS level by 0.9-fold, while CeO_2_ NPs at 25 and 50 mg L^−1^ increased the ROS level by 1.4-fold and 1.5-fold when compared to the control (Figure 4f). In System 2, the gene expression for mutagenesis repair in the SOS response (*umuc*) remained unchanged in the presence of 0.5 and 10 mg L^−1^ of CeO_2_ NPs, while 50 mg L^−1^ CeO_2_ NPs induced the expression of *umuc* by 3.2-fold (Figure 4g). Moreover, the cell membrane permeability decreased by 0.9-fold in the presence of 0.5 mg L^−1^ CeO_2_ NPs (Figure 4h). In accordance, the expression of genes related to membrane permeability (*ompA* and *ompC*) decreased by 0.6–0.7 fold after exposure to 0.5 mg L^−1^ CeO_2_ NPs (Figure 4g). Bacterial outer membrane proteins (OMP) including OmpA and OmpC are associated with membrane permeability and gene transfer, contributing to the passive diffusion of molecules smaller than 500–600 Da [48,49]. Unlike System 2, the cell membrane permeability in System 3 remained unchanged after exposure to CeO_2_ NPs at 0.5–25 mg L^−1^, and a significant increase (by 1.1-fold) was observed in the presence of 50 mg L^−1^ CeO_2_ NPs (Figure 4i). Similar regulation of gene expression for cell membrane permeability (*ompA*, *ompC* and *umuC*) was consistently detected (Figure 4j). The increase in membrane permeability after exposure to 50 mg L^−1^ CeO_2_ NPs could be attributed to the self-aggregation of CeO_2_ NPs and their adsorption to cell surface, leading to an increase in the intracellular ROS content (Figure 4j,k). Hence, incubation of competent cells with CeO_2_ NPs at relatively low concentrations (0.5 mg L^−1^) could reduce the membrane permeability, inhibiting the transformation of free plasmids (Figure 4a). However, it seems that the ROS level and membrane permeability did not contribute to the inhibited transformation by pre-incubation of plasmids with CeO_2_ NPs. In addition to the binding of CeO_2_ NPs, effects of CeO_2_ NPs on the transformation process were expected.

### 3.4. Expression of Transformation-Related Genes Altered by CeO_2_ NPs

**Figure 5:**
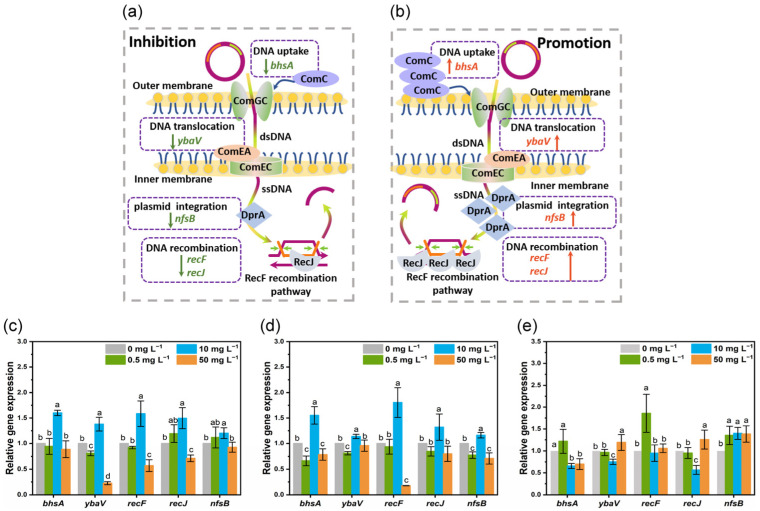
Effect of CeO_2_ NPs on the gene expression related to transformation. Schematic plot for plasmid-borne ARGs uptake and integration mediated by CeO_2_ NPs (**a**,**b**). Relative expression of transformation-related genes regulated by CeO_2_ NPs (0, 0.5, 10, and 50 mg L^−1^) in System 1 (**c**), System 2 (**d**), and System 3 (**e**). Lowercase letters represent the statistical significance among different concentrations of CeO_2_ NPs.

DNA uptake and integration are two primary processes involved in transformation (Figure 5a,b) [50]. ComGC is a cell surface-localized protein for DNA binding during transformation that is processed by ComC [51]. The expression of *bhsA* encoding protein ComC had been up-regulated significantly (by 1.6-fold) in the presence of 10 mg L^−1^ CeO_2_ NPs (System 1 and 2) (Figure 5c,d). However, CeO_2_ NPs at 0.5 and 50 mg L^−1^ either significantly down-regulated (System 2, by 0.6–0.8 folds) or did not affect (System 1) the expression of *bhsA* (Figure 5c,d). Pre-incubation of plasmids with 10 and 50 mg L^−1^ CeO_2_ NPs (System 3) significantly inhibited the expression of *bhsA* by 0.7–0.8 folds (Figure 5e). ComGC is a major pilin of T4P-related structure for conveying exogenous DNA to the dsDNA receptor ComEA [50,52]. The expression of *ybaV* encoding protein ComEA had been significantly down-regulated (by 0.8–0.9 folds) after exposure to 0.5 mg L^−1^ CeO_2_ NPs (System 1 and 2, Figure 5c,d). However, 10 mg L^−1^ CeO_2_ NPs elevated the expression of *ybaV* in System 1 and 2 (up to 1.4-fold and 1.1-fold, respectively) (Figure 5c,d). In System 3, CeO_2_ NPs at 10 or 50 mg L^−1^ down- or up-regulated the *ybaV* expression by 0.7- or 1.2-fold (Figure 5e). Proteins encoded by the ComG operon and ComC are essential for DNA binding to competent cells, providing access for ComEA to exogenous DNA [52]. ComEA plays a vital role in the transfer of transforming DNA into the DNA channel and in controlling the rate of DNA uptake [53]. Regardless of the different systems, it seems that the transformation frequency remained unchanged if the expression of either *bhsA* or *ybaV* was unaffected (despite 50 mg L^−1^ for System 1), while inhibition or stimulation of transformation occurred only if both genes were simultaneously down- or up-regulated (Figure 5a,b). Altogether, pre-treatment of competent cells or plasmids with CeO_2_ NPs at relative low concentrations (0.5 or 10 mg L^−1^) down-regulated the gene expression for DNA binding to the membrane surface, thus inhibiting the transformation (Figure 5a); conversely, treating a mixture of competent cells and plasmids with CeO_2_ NPs at 10 mg L^−1^ enhanced the DNA uptake process.

The retraction of pseudopilus facilitates the contact of double-stranded DNA (dsDNA) with protein ComEA, after which one strand of DNA is degraded by EndA and the remaining single-stranded DNA (ssDNA) is translocated into cytoplasm through the ComEC transmembrane channel [50,54]. The internalized ssDNA is bound by ssDNA-binding protein DprA (encoded by gene *nfsB*), which is essential for the formation of duplex circular plasmid and homologous recombination of incoming ssDNA [54,55,56]. The expression of *nfsB* was up-regulated in the presence of 10 mg L^−1^ CeO_2_ NPs for all three systems (Figure 5c–e), while CeO_2_ NPs at 0.5 and 50 mg L^−1^ led to an opposite regulation of *nfsB* expression in System 2 (down-regulation by 0.8- and 0.7-fold, respectively) and System 3 (up-regulation by 1.3- and 1.4-fold, respectively) (Figure 5d,e). Mutation of *nfsB* in *Helicobacter pylori* inhibited the uptake of plasmids, significantly reducing the transformation frequency (over 90%); additionally, *Deinococcus radiodurans* (*D. radiodurans*) exhibited a 21-fold reduction of plasmid transformation frequency in the absence of the DprA protein [57,58]. Thus, although the expression of *ybaV* remained unchanged in System 2 (50 mg L^−1^ CeO_2_ NPs), the down-regulation of *nfsB* and *bhsA* may contribute to the decreased transformation (Figure 5d). DNA replication is known to be closely related to the recF pathway of recombination [59]. RecJ, a member of DHH family of proteins, is the only 5′ → 3′ exonuclease involved in the RecF recombination pathway [60]. The frequency of plasmid recombination decreased with the mutation of *recJ* in *E. coli* strains [61]. The expressions of *recF* and *recJ* were up-regulated in *E. coli* cells exposed to 10 mg L^−1^ CeO_2_ NPs (System 1 and 2) (Figure 5c,d), whereas significant down-regulation (0.6- and 0.7-fold) was observed in cells exposed to 50 mg L^−1^ CeO_2_ NPs in System 1 (Figure 5c). Thus, pre-exposure of competent cells with CeO_2_ NPs (0.5 or 50 mg L^−1^) in both System 1 and 2 inhibited the DNA uptake and integration (Figure 5a,b), with System 2 being more sensitive to the CeO_2_ NPs. Compared to the plasmids, the positively charged CeO_2_ NPs were more likely to be attracted to the cell membrane, leading to an increased internalization of NPs and up-regulation of functional genes. In System 3, CeO_2_ NPs induced the expression of genes involved in DNA recombination regardless of different concentrations (Figure 5e). However, only 10 mg L^−1^ CeO_2_ NPs inhibited the transformation while 0.5 and 50 mg L^−1^ did not show any impact. Similarly, CeO_2_ NPs at 10 mg L^−1^ exhibited a high binding affinity to DNA and a strong inhibitory effect on the gene expression for DNA replication [62]. In addition, the double mutation of *dprA* and *recF* completely abolished DNA transformation in *D. radiodurans* [58]. On this basis, we speculated that the simultaneous down-regulation of *recF* and *nfsB* in System 2 (50 mg L^−1^) might have contributed to the significant reduction in transformation. CeO_2_ NPs (particularly 10 mg L^−1^) may inhibit the transformation of eARGs by suppressing the replication of DNA. However, for System 1 and 2, a high concentration of CeO_2_ NPs (50 mg L^−1^) was required to generate same inhibiting effect on DNA replication, thereby reducing the transformation frequency.

## 4. Conclusions

ARGs are persistent in natural aquatic and soil environments. Transformation enables the evolution of competent bacteria into resistant species. Nanomaterials exhibit unique bioactivities due to their nanoscale size, high specific surface area, and precise nanostructure, significantly influencing the interactions between materials and biological systems to regulate cellular behaviors and functions [63]. Here, we have shown the potential of CeO_2_ NPs for controlling the transformation process. Particularly, pre-incubation of plasmids with CeO_2_ NPs at 10 or 25 mg L^−1^ could effectively inhibit the transformation by reducing plasmid availability, inhibiting DNA replication, and down-regulating the gene expression of DNA uptake. Incubation of competent cells with CeO_2_ NPs at a low concentration (0.5 mg L^−1^) can inhibit the transformation via decreasing the ROS content and cell membrane permeability, down-regulating the expression of genes related to DNA uptake and processing; CeO_2_ NPs at relatively high concentrations (10 and 25 mg L^−1^) led to an opposite effect. Still, incubation of CeO_2_ NPs with plasmids and competent cells in complex environmental conditions, such as soil and aquatic environments, is needed to make this nano-strategy more practical. Our findings provide insights on the application of nanomaterials for regulating transformation of extracellular ARGs.

## Data Availability

The data presented in this study are available on request from the corresponding author.

## Supplementary Materials

The following supporting information can be downloaded at: https://www.mdpi.com/article/10.3390/nano13060969/s1, Figure S1: The morphology and structure of CeO2 NPs characterized by TEM and XRD.; Figure S2: Agarose gel electrophoresis of PCR amplicons of recipient E. coli cells and transformants from System 1–3.; Figure S3: Transformation efficiency of pAC plasmids in E. coli cells in System 1–3.; Table S1: The hydrodynamic diameter and zeta-potential of CeO2 NPs.; Table S2: PCR primers.; Table S3: Particle content and size distribution of CeO2 NPs internalized in E. coli cells.; Table S4: Hydrodynamic diameter of CeO2 NPs and plasmids exposed to CeO2 NPs.
