# Supplementary material for: CeO2 Nanoparticles-Regulated Plasmid Uptake and Bioavailability for Reducing Transformation of Extracellular Antibiotic Resistance Genes
Source: Nanomaterials (Basel). 2023 Mar 8;13(6):969. doi: 10.3390/nano13060969 (PMC10053900; doi:10.3390/nano13060969)
Supplement: Supplementary file 1 [file nanomaterials-13-00969-s001.zip › nanomaterials-2251489-supplementary.pdf]

Supplementary Materials

CeO_2 Nanoparticles-Regulated Plasmid Uptake and Bioavailability for Reducing Transformation of Extracellular Antibiotic Resistance Genes

Yinuo Xu [1,2], Hao Du [1,2], Chuanxi Wang [1,2], Le Yue [1,2], Feiran Chen [1,2,*] and Zhenyu Wang [1,2,3]

1 Institute of Environmental Processes and Pollution Control, ~~and~~ School of Environmental and Civil Engineering, Jiangnan University, Wuxi 214122, China; 6201403016@stu.jiangnan.edu.cn (Y.X.); 6201405039@stu.jiangnan.edu.cn (H.D.); wangcx2018@jiangnan.edu.cn (C.W.); leyue@jiangnan.edu.cn (L.Y.); wang0628@jiangnan.edu.cn (Z.W.)
2 Jiangsu Engineering Laboratory for Biomass Energy and Carbon Reduction Technology, Jiangnan University, Wuxi 214122, China
3 Jiangsu Collaborative Innovation Center of Technology and Material of Water Treatment, Suzhou University of Science and Technology, Suzhou 215009, China
* Correspondence: chenfeiran@jiangnan.edu.cn

List of Supplementary Material

Figure S1. The morphology and structure of CeO_2 NPs characterized by TEM and XRD.

Figure S2. Agarose gel electrophoresis of PCR amplicons of recipient *E. coli* cells and transformants from System 1-3.

Figure S3. Transformation efficiency of pAC plasmids in *E. coli* cells in System 1-3.

Table S1. The hydrodynamic diameter and zeta-potential of CeO_2 NPs.

Table S2. PCR primers.

Table S3. Particle content and size distribution of CeO_2 NPs internalized in *E. coli* cells.

Table S4. Hydrodynamic diameter of CeO_2 NPs and plasmids exposed to CeO_2 NPs.

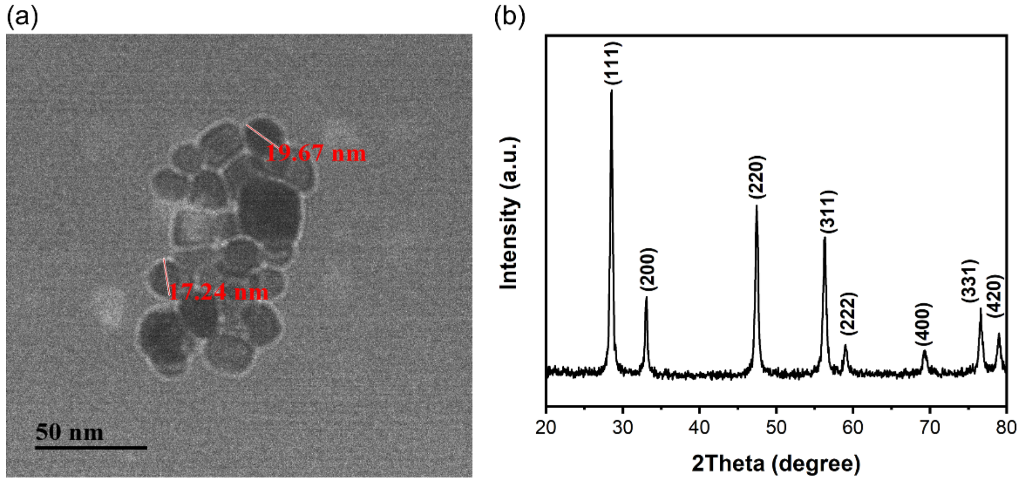

Figure. S1. The morphology and structure of CeO_2 NPs characterized by TEM and XRD.

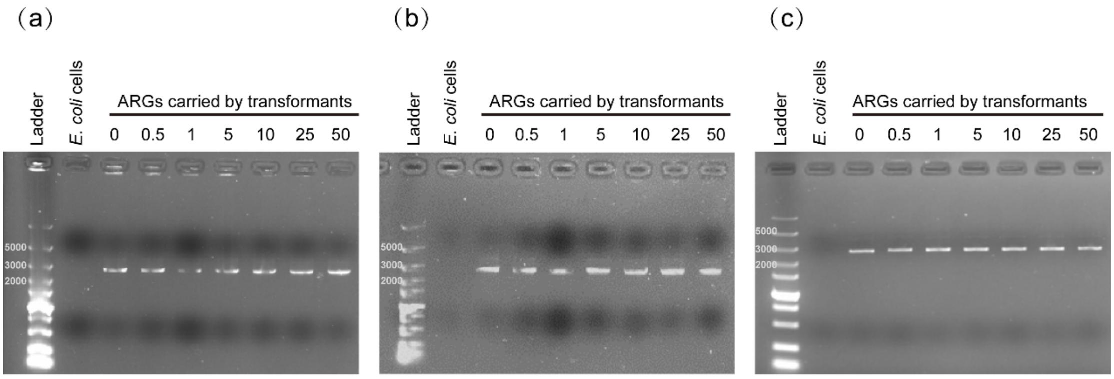

Figure. S2. Agarose gel electrophoresis of PCR amplicons of recipient *E. coli* cells and transformants from System 1 (a), System 2 (b), and System 3 (c).

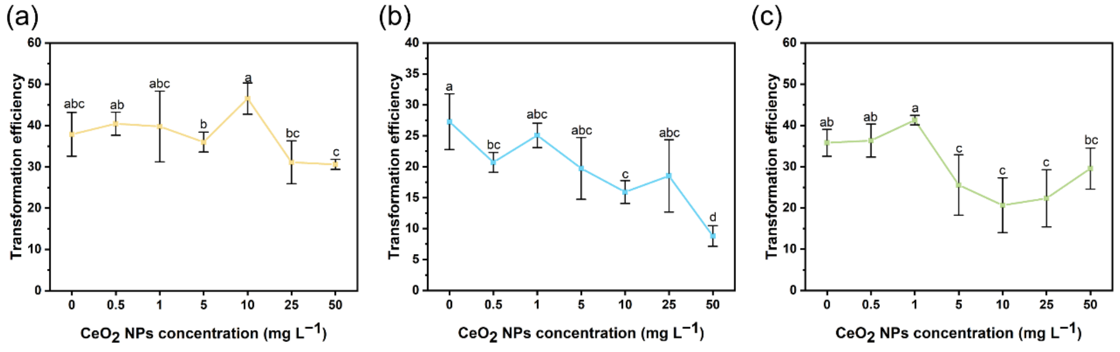

Figure. S3. Transformation efficiency of pAC plasmids in *E. coli* cells in System 1 (a), System 2 (b), and System 3 (c).

Table S1. Hydrodynamic diameter and zeta-potential of CeO_2 NPs.

Concentrations ($mg\ L^{-1}$)	Hydrodynamic diameter (nm)	Zeta-potential (mV)
10	1173.5±69.6	24.7±0.5
25	1117.9±143.6	23.9±0.8
50	1278.7±64.6	24.2±1.8

Table S2. PCR primers.

Gene name	FW-Primer	RW-Primer
pAC-mch	GAAATCTACTGGTATTGTACG TATGGTATCAAAAGGAGAGGA AGA	CGCAGTCACCAAAACTTGTCC TTTCAGTTTAGCCT
ompA	TGAGCCTGGGTGTTTCCTA	CAGAGCAGCCTGACCTTCC
ompC	AAGTAGTAGGTAGCACCAAC ATCA	GGGCGAACAAAGCACAGAA
umuC	AAACTGCTGACGCCCACTC	GCGACTCCCTGACTGAAGA AA
bhsA	GCGGCGATTTTAAGCTCCAT	CCAGATTTGTCCCCGCGTTA
ybaV	CCTGTGCCGGAATGTCTCAT	GCCGGTACTGCTGCTTTACT
recF	GAATGACGCGACCAATCTGC	CGCTTGTTGATCCGCGATTT
recJ	GACCGGAACCTTTCAGCGTA	CTGACCCTGTGCGAGAAACT
nfsB	GATGTTTTGCGGCAGACGAG	CCCATCGAAGGTTTTGACGC

Table S3. Particle content and size distribution of CeO_2 NPs internalized in *E. coli* cells

	Concentrations (mg L^{-1})	Size (nm)	Content of cellular Ce particles (particles mg^{-1})
System 1	0	38.4±0.6 ^{b}	7270.4±683.1 ^{c}
	0.5	38.8±0.9 ^{b}	7598.9±680.4 ^{c}
	10	42.7±6.2 ^{ab}	37209.6±375.3 ^{b}
	50	45.0±3.3 ^{a}	59598.4±10984.2 ^{a}
System 2	0	38.3±2.9 ^{a}	8768.2±1607.8 ^{b}
	0.5	41.7±6.9 ^{a}	9889.8±1354.4 ^{b}
	10	42.7±2.0 ^{a}	62475.8±8699.1 ^{a}
	50	41.8±3.3 ^{a}	82151.0±12575.9 ^{a}
System 3	0	32.6±2.4 ^{a}	7928.6±969.0 ^{c}
	0.5	34.7±3.1 ^{a}	7282.1±1183.9 ^{c}
	10	35.5±2.9 ^{a}	28317.2±2060.0 ^{b}
	50	37.2±2.5 ^{a}	50184.4±5645.1 ^{a}

Lowercase letters represent the statistical significance among different concentrations of CeO_2 NPs.

Table S4 Hydrodynamic diameter of CeO_2 NPs and plasmids exposed to CeO_2 NPs.

	Concentrations (mg L^{-1})	Hydrodynamic diameter (nm)
CeO_2 NPs suspensions (2 h)	10	1177.2±96.7 ^{b}
	25	1140.7±75.3 ^{b}
	50	2349.4±243.1 ^{a}
Plasmids exposed to CeO_2 NPs (2 h)	10	1386.3±82.9 ^{b}
	25	1437.2±146.5 ^{b}
	50	2046.4±173.9 ^{a}

Lowercase letters represent the statistical significance among different concentrations of CeO_2 NPs.
